# Redesigned Skip-Network for Crowd Counting with Dilated Convolution and Backward Connection

**DOI:** 10.3390/jimaging6050028

**Published:** 2020-05-02

**Authors:** Sorn Sooksatra, Toshiaki Kondo, Pished Bunnun, Atsuo Yoshitaka

**Affiliations:** 1 School of Information and Communication Technology, Sirindhorn International Institute of Technology, Thammasat University, Pathum Thani 12120, Thailand; tkondo@siit.tu.ac.th; 2 School of Information Science, Japan Advanced Institute of Science and Technology, Ishikawa 923-1211, Japan; ayoshi@jaist.ac.jp; 3 National Electronic and Computer Technology Center, National Science and Technology Development Agency, Pathum Thani 12120, Thailand; pished.bunnun@nectec.or.th

**Keywords:** surveillance system, crowd counting, regression-based approach, skip connection, dilated convolution

## Abstract

Crowd counting is a challenging task dealing with the variation of an object scale and a crowd density. Existing works have emphasized on skip connections by integrating shallower layers with deeper layers, where each layer extracts features in a different object scale and crowd density. However, only high-level features are emphasized while ignoring low-level features. This paper proposes an estimation network by passing high-level features to shallow layers and emphasizing its low-level feature. Since an estimation network is a hierarchical network, a high-level feature is also emphasized by an improved low-level feature. Our estimation network consists of two identical networks for extracting a high-level feature and estimating the final result. To preserve semantic information, dilated convolution is employed without resizing the feature map. Our method was tested in three datasets for counting humans and vehicles in a crowd image. The counting performance is evaluated by mean absolute error and root mean squared error indicating the accuracy and robustness of an estimation network, respectively. The experimental result shows that our network outperforms other related works in a high crowd density and is effective for reducing over-counting error in the overall case.

## 1. Introduction

In recent years, crowd counting has an important role in a wide range of applications (e.g., video surveillance, traffic monitoring [1], public safety, urban planning [2], and so on). These applications have the same goal of collecting statistical data (the number of objects). Then, this data is utilized in further areas (e.g., crowd management, behavior observation, and so on). Therefore, crowd counting must be accurate enough for a practical usage. With the increase of diversity, the crowd density is dynamically varied in different locations and times. The video surveillance system usually captures a large or small number of people at any time. This circumstance shows that crowd counting must be able to count objects in different crowd density. On the other hand, the scaling problem should be considered because of the different adjustments in camera viewpoints and varied sizes of objects.

**Figure 1:**
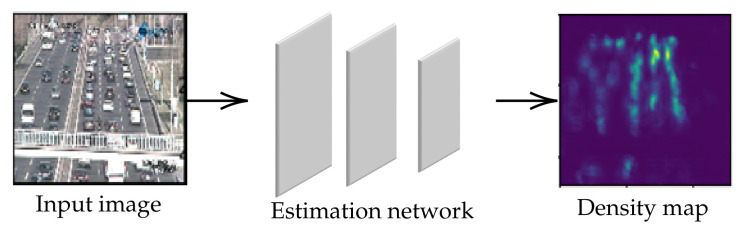
A general flowchart of crowd counting using deep learning-based technique.

It is well known that a detection-based technique is ineffective for counting several objects from the crowd image because of occlusion problems. Therefore, numerous researchers have relied on a regression-based technique. This technique emphasizes counting-by-density algorithms to predict the crowd density, called ’density map’. As we have already known, state-of-the-art approaches have utilized a deep learning-based technique with Convolutional Neural Network (CNN) to design an estimation network and obtain a density map as shown in Figure 1. To solve the scaling problem, estimation networks from previous studies have trended to implement with several image resolutions and convolutional layers (Conv layers) for handling various object scales. However, their valuable information could be lost by resizing crowd images and their results were limited by the network configuration in different datasets. On the other hand, skip connections have been added to the estimation network for reusing feature maps, rather than building other networks. This technique is called ’skip-network’ which is effective only on objects with large scales because high-level feature maps are only emphasized.

In this paper, we propose a novel method for implementing an estimation network with skip-network. Instead of using a normal skip connection or a forward connection, we propose a backward connection which extracts feature maps from the deeper layer to a shallow layer. It helps the shallow layer to recognize the characteristic of the target in advance. False positives can be reduced before formulating the density map. In our expectation, objects with small and large scales are corresponded to shallow and deep layers, respectively. Considering the quality of a density map, feature maps in every layer should have the same resolution to prevent information loss from adjusting their resolutions. Then, pooling layers are replaced with a dilated Conv layer. The receptive field with a dilated Conv layer grows exponentially while keeping the resolution of a feature map [3]. In addition, fewer model parameters are required without resizing feature maps. Therefore, our contributions are summarized as follows:

- The proposed network utilized the backward connection for passing high-level features from deeper layers to shallower layers and reducing false positives in crowd counting.
- Dilated convolution is introduced in skip-network to increase the receptive field sizes while keeping the information of high-level features for a feature map integration in the skip connection.

## 2. Related Work

**Figure 2:**
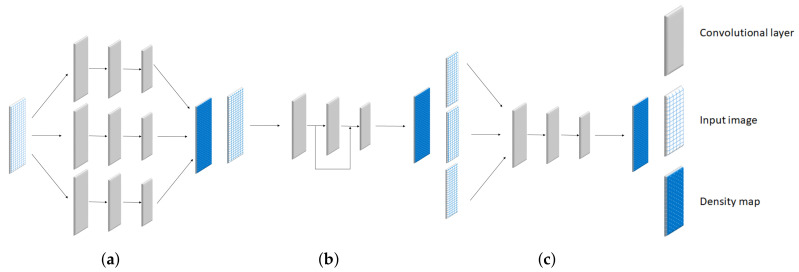
Illustrations of network architectures of (**a**) multi-column network, (**b**) skip-network, and (**c**) multi-scale network.

Over the past decade, CNN has been utilized for designing estimation networks in crowd counting. Their networks are categorized into 3 types, which are multi-column network, skip-network, and multi-scale network as shown in Figure 2a–c, respectively. Their features are described as follows:

### 2.1. Multi-Column Network

The multi-column network is the first technique for handling the scaling problem. This network consists of multiple CNNs extracting features in different scales as shown in Figure 2a. Their results from each network were merged to improve the counting performance in various scales [4,5]. The network architecture was designed by adjusting its kernel size for a specific object size or scale. Instead of relying on the various kernels, the number of the Conv layer was considered by [6]. On the other hand, some related studies have trended to design a gate network for selecting the most optimal CNN in [2,7]. These multi-column methods partially solve the scaling problem, however, their network architectures consist of a large number of trainable parameters. In addition, their achievements were limited by the number of networks and the size of the receptive field, depending on the scales of objects in the dataset.

### 2.2. Skip-Network

In recent years, the skip-network has been utilized in an estimation network. It reused the intermediate layers for extracting various features of objects in different sizes. Skip-network architecture is illustrated in Figure 2b, where it makes a connection between low and high-level features from shallow to deep layers, respectively. The key concept of skip-network is to build various receptive fields for handling objects in different sizes [8,9]. It is similar to the multi-column network, but fewer model parameters are involved. Their counting performance was improved by adding a multi-scale unit [10]. This unit is a structure of subset in skip-network which helps to increase more receptive fields in one Conv layer. In addition, a Scale Focus Module (SFM) was inserted to enhance the local feature in different scale. Even though the trainable parameter can be reduced, only high-level features are emphasized by extracting low-level features from shallow layers. It means that the object with a large scale is not effective by this technique.

### 2.3. Multi-Scale Network

In the multi-scale network, several crowd images are fed into an estimation network to learn the characteristic of a crowd image in different conditions as shown in Figure 2c. A deep, single-column, and fully connected network was utilized to predict crowd density maps. This method addresses the issues of scale and perspective changes by feeding multiple scales of testing images into the estimation network during the prediction phase [11]. On the other hand, the dataset was categorized by the camera viewpoint. The input image in the different categories was trained to optimize an estimation network [1]. Similar to a multi-column network, their performance is limited to a variation of an input image in a different dataset.

This paper discusses a redesigned skip-net emphasizing high-level features represented in objects with large scales by using the small number of input data and model parameters. The backward connection with dilated convolution is proposed to optimize the weight in a shallow layer for objects with small scales and reduce the counting error from resizing.

## 3. Density Map for Object Counting

In general, a crowd image is annotated as a matrix containing the central location or the position of the object. The matrix containing these annotations is called ’position map’ (H). This position map is considered as a sum of the delta function (δ) calculated in Equation (1), where (x,y) is vertical and horizontal coordinate, respectively, and $\left(x_{i},y_{i}\right)$ is vertical and horizontal positions for the *i*th object, respectively. As far as we are aware, the ground truth or an actual density map (*D*) was prepared by a convolution between *H* and Gaussian kernel (G) formulated in Equation (2), where $\sigma_{i}$ is a spread parameter of the *i*th object.

(1) $$H\left(x,y\right)=\sum_{i=1}^{N}\delta \left(x-x_{i},y-y_{i}\right)$$

(2) $$D\left(x_{i},y_{i}\right)=H\left(x_{i},y_{i}\right)*G\left(\sigma_{i}\right)$$

In this paper, we have followed the technique from [4] to calculate an actual density map. Since each region in a crowd image has a different crowd density, $\sigma_{i}$ should be varied in each location of the crowd image. The crowd density could be related to the distance between two object positions. For each object in given images, the distance to its *k* nearest neighbor of the *i*th object is denoted by { $d_{1}^{i},d_{2}^{i},d_{3}^{i},...,d_{k}^{i}$ }. Therefore, $\sigma_{i}$ is evaluated from an average distance between objects, which are inversely proportional to crowd density, as depicted in Equation (3),

(3) $$\sigma_{i}=\frac{\beta }{k}\sum_{j=1}^{k}d_{j}^{i}$$

where β is a constant factor to control $\sigma_{i}$. In other words, each location has its kernel ($\sigma_{i}$) which is adaptive to crowd density. In the experiment, β and *k* were set as 0.2 and 4, respectively. As a result, the density map can be used for calculating the number of objects or an actual count ($C_{A}$) from Equation (4), where $S_{x}$ and $S_{y}$ are the vertical and horizontal dimensions of crowd images, respectively.

(4) $$C_{A}=\sum_{x=1}^{S_{x}}\sum_{y=1}^{S_{y}}D\left(x,y\right)$$

## 4. Estimation Network Architecture

As mentioned in Section 2, an estimation network plays an important role in crowd counting by the regression-based method. This paper presents the skip-network with the backward connection for crowd counting, where network architectures are described as follows:

### 4.1. Backbone Network

**Figure 3:**
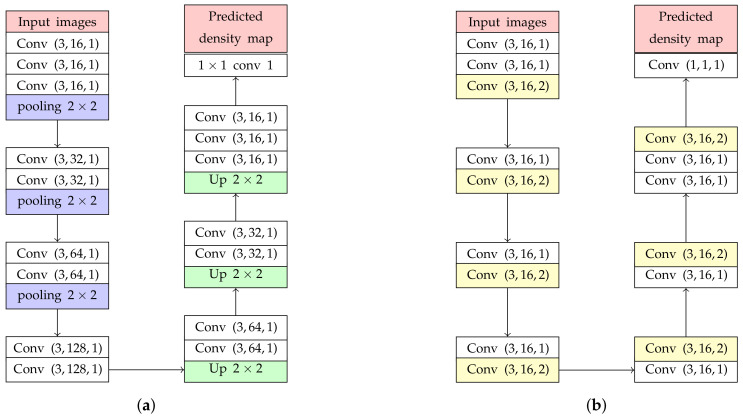
The backbone network architecture modified by (**a**) adding pooling and up-sampling layers and (**b**) constructing only convolutional layers (Conv layers).

The backbone network is modified from our previous study [12]. Its sequences of Conv layers were utilized in this paper as shown in Figure 3a, where Conv (3,16,1) is represented as a Conv layer with 3×3 kernel size, the number of filter = 16, and a dilated rate = 1. Our previous network is divided into two parts, encoder and decoder. The encoder consists of Conv and pooling layers utilized for extracting low-level features. Furthermore, the decoder consists of Conv layers and up-sampling layer (Up) for recovering information lost by pooling layers and extracting high-level features. To limit the network complexity, kernel sizes of all Conv layers were set as the smallest value (3×3). In addition, we found that a stack of 3×3 Conv layers, called ’Conv block’, gives higher counting performance in prediction and classification [13].

The previous studies [14] found that resizing feature maps, with pooling and up-sampling layers, may cause an error in object counting calculated in (4) because valuable information might be lost. Then, only the Conv layer should be employed in the backbone network without resizing their resolutions. However, the receptive field size is gradually increased by utilizing the sequence of Conv layers only. It means that more parameters and layers are required for implementing an estimation network. To solve this problem, pooling and up-sampling layers are replaced with dilated convolution represented as yellow layers in Figure 3b. The dilation convolution is defined in Equation (5),

(5) $$y\left(m,n\right)=\sum_{i=1}^{M}\sum_{j=1}^{N}x\left(m+r\times i,n+r\times j\right)w\left(i,j\right)$$

where (m,n) is the location in terms of vertical and horizontal coordinates of the feature map and y(m,n) is the output of a dilated Conv layer from their input features (x(m,n)) and weights (w(m,n)) with the height and width of *M* and *N*, respectively. The parameter *r* is the dilation rate, where the normal convolution are assigned with r=1. The dilated Conv layer is able to increase the size of receptive field exponentially without resizing. In addition, the experiment from [14] has proved that the quality of the density map is improved by using an estimation network with only normal and dilated convolutions. Based on our experiment, a backbone network with a dilated rate = 2 is selected because it gives the most optimal performance of the density map prediction. To keep a feature map resolution, all Conv layers use zero-padding to maintain the previous size.

### 4.2. Backward Connection

As mentioned in Section 2, this paper confronts a scaling problem in the crowd counting. In our hypothesis, the crowd image generally consists of objects with small scales or highly overlapping regions which are represented as low-level features in shallow layers. Instead of relying a forward connection in the previous skip-network, this paper implemented an estimation network with a backward connection which has the following advantages:

- Shallower layers can recognize the characteristic of a feature map from the deeper layer to emphasize low-level features, which can be considered as the main features of crowd images.
- Since CNNs have a hierarchical network architecture, the performance for crowd counting with large object can be improved.

**Figure 4:**
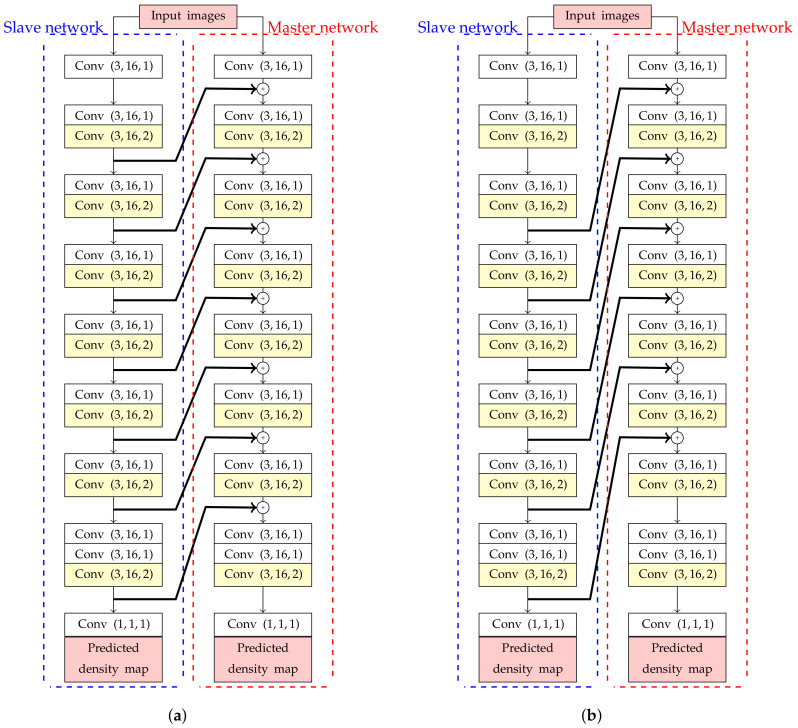
The estimation network architectures of backbone networks with backward connections, consisting of master (right) and slave networks (left) with (**a**) K=1 and (**b**) K=2 backward connections.

To design an estimation network with backward connections, high-level features need to be extracted before passing information into shallow layers. However, if these high-level features are simply added into shallow layers, it will create a cyclic loop in an estimation network. To solve this problem, the master and slave networks were designed in this study as shown in Figure 4. These two networks have the same sequence of Conv layers, but their weights are optimized independently. Even though both master and slave networks are optimized by the same loss function, predicted density from a master network is only extracted for calculating the number of object.

In the next step, a pair between deeper and shallower layers should be analyzed to optimize the estimation network performance. Let *K* be the number of skipped Conv blocks in an estimation network. Then, the the general formula for adding *K* backward connections as formulated in (6),

(6) $$Y_{L}^{m}=F_{L}^{m}\left(X_{L}^{m}\right)⊕F_{L+K}^{s}\left(X_{L+K}^{s}\right)$$

where ($F_{L}^{s}$, $F_{L}^{m}$) are the *L*th of Conv blocks in slave (’*s*’) and master (’*m*’) networks with ($X_{L}^{s}$, $X_{L}^{m}$) as their input feature maps, respectively, and $Y_{L}^{m}$ is the output feature map from $F_{L}^{m}$. The examples of backbone network with (K=1 and 2) backward connections are shown in Figure 4a and Figure 4b, respectively. In addition, all feature maps in each Conv blocks have the same dimension because of dilated convolution. Any convolutional transformations to adjust dimension between lower and higher-level feature maps is not required in this paper.

## 5. Training Method

In this section, specific detail for training an estimation network with backward connections is provided. Weight optimization and pre-processing of input data are concerned as follows:

### 5.1. Optimization of an Estimation Network

The trainable parameters of an estimation network were optimized by using a loss function. During training, Euclidean distance was calculated to measure the loss function (*L*) between the predicted and actual density maps (*D*). Even though an actual count could be set as the final output of an estimation network, we decided to select an actual density map instead because of the following reasons:

- More spatial information is preserved in a density map. Even though a crowd image contains the same number of objects, the pattern or spatial distribution can be different. Therefore, an estimation network can be optimized or handled for various conditions of crowd images.
- In the optimization process, the size of an object or Gaussian kernel is suitable for learning on CNN. The spatial kernel in Conv layers can be adapted to different sizes whose perspective effect varies significantly. Thus, spatial kernels are more semantic meaningful, and consequently, they improve the accuracy of crowd counting.

As described in the previous section, the proposed estimation network consists of two networks, slave and master networks. Their parameters of slave and master networks were optimized by their loss functions in Equations (7) and (8), respectively,

(7) $$L^{s}\left(\Theta^{s}\right)=\frac{1}{2N}\sqrt{\sum_{t=1}^{N}{\left(R_{t}^{s}\left(I_{t},\Theta^{s}\right)-D_{t}\right)}^{2}}$$

(8) $$L^{m}\left(\Theta^{m}\right)=\frac{1}{2N}\sqrt{\sum_{t=1}^{N}{\left(R_{t}^{m}\left(I_{t},\Theta^{m}\right)-D_{t}\right)}^{2}}$$

where ($\Theta^{s}$, $\Theta^{m}$) and ($L^{s}$, $L^{m}$) are trainable parameters and loss functions of slave (’*s*’) and master (’*m*’) networks, respectively. *N* is the number of training images. $I_{t}$ is a crowd image with an index ’*t*’. ($R_{t}^{s},R_{t}^{m}$) and ($D_{t}^{s},D_{t}^{m}$) are predicted and actual density maps with index ’*t*’ in the slave (’*s*’) and master (’*m*’) networks, respectively. Then, the total loss was calculated by Equation (9) for evaluating validation images, where $\Theta^{t}$ is the total trainable parameters of an estimation network.

(9) $$L^{t}\left(\Theta^{t}\right)=\sqrt{{\left(L^{m}\left(\Theta^{m}\right)\right)}^{2}+{\left(L^{s}\left(\Theta^{s}\right)\right)}^{2}}$$

### 5.2. Data Augmentation

**Figure 5:**
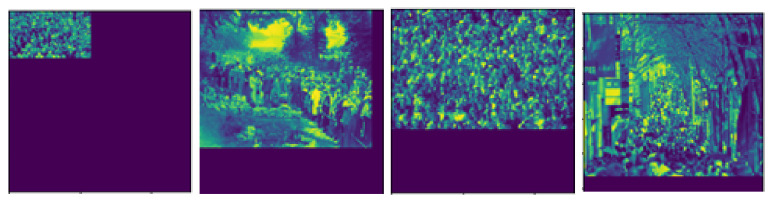
The examples of regions located in the boundary of images (heat maps) after zero padding.

Image resolution should concern with network optimization. Since the training images were obtained from several sources, their image resolutions might be different. Therefore, the image must be resized in the dimension before feeding into an estimation network. However, the object positions may be changed after resizing images. To solve this problem, crowd images were divided into several regions, each of which consists of size 256×256 pixels. For regions located at the boundary of images, zero padding was applied to increase the image size as shown in Figure 5. Then, the resolutions of training and testing images are normalized into 256×256 pixels.

## 6. Experimental Results

The experiment was evaluated on 3 different datasets with the the same evaluation metric (Section 6.1). In our experimental setup, an actual density map derived from Equation (2) was calculated as the ground truth of an estimation network. The estimation network was optimized by Equation (9) with learning rate = $1\times 10^{-4}$.

### 6.1. Evaluation Metric

Followed by the existing methods, the evaluation metric utilizes Root Mean Squared Error (RMSE) and Mean Absolute Error (MAE) calculated by Equations (10) and (11), respectively.

(10) $$RMSE=\sqrt{\frac{1}{N}\sum_{t=1}^{N}{\left(R_{t}^{m}-D_{t}\right)}^{2}}$$

(11) $$MAE=\frac{1}{N}\sum_{t=1}^{N}\left|R_{t}^{m}-D_{t}\right|$$

where *N* is the number of testing images. $R_{t}^{m}$ and $D_{t}$ are predicted and actual density maps from a master (’*m*’) network at a frame with index ’*t*’, respectively. MAE is more significant than RMSE for counting performance because RMSE is relative as the variance associated with the frequency distribution of error magnitudes also increases while MAE describes average error alone. RMSE is more difficult to tease out and understand. However, RMSE is utilized for evaluating the robustness of an estimation network [15]. Even though the predicted density was utilized for optimization, predicted count ($C_{P}$) calculated by Equation (12) was analyzed.

(12) $$C_{P}=\sum_{x=1}^{S_{x}}\sum_{y=1}^{S_{y}}R^{m}\left(x,y\right)$$

### 6.2. Crowd Counting Dataset

To test our crowd counting, we decided to evaluate our estimation network with various datasets and targets. The human counting was evaluated by ShanghaiTech and UCF_CC_50 datasets. On the other hand, this proposed method was also evaluated for non-human counting by using the TRANCOS dataset (vehicle counting). Their information is described as below:

#### 6.2.1. Shanghaitech Dataset

It is the dataset for crowd counting, targeting on human in the crowd image. This large-scale crowd counting dataset was introduced by [4]. It contains 1198 annotated images, with a total of 330,165 people and the central coordinates of their heads. This dataset consists of two parts as follows:

- Part A: There are 300 training and 182 testing images collected randomly crawled from the internet.
- Part B: There are 400 training and 316 testing images taken from crowded scenes on Shanghai streets.

#### 6.2.2. UCF _CC _50 Dataset

Their crowd images were published by [16]. This dataset consists of 50 crowd images obtained from the internet. It is a very challenging dataset because The number of images is limited and the crowd count of the image changes dramatically. The total number of head (annotations) is 63,974 for these 50 crowd images. The headcount range between 94 and 4543 with an average of 1280 individuals per image. Since this dataset provides a small number of crowd images, this dataset was evaluated by using estimation networks trained by ShanghaiTech dataset (Part A and Part B).

#### 6.2.3. TRANCOS Dataset

This is a well-known dataset, for a vehicle counting, distributed by [17]. It represents crowd images with various traffic density. This dataset contains different 1244 traffic images captured in various locations. Their crowd images were divided into training, testing, and validation images, selected by the dataset owner. The training, testing, and validation samples contain 403, 421, and 420 crowd images, respectively.

### 6.3. Ablations on Shanghaitech Part A

As described in Section 4.2, the estimation networks with various *k* backward connections are formulated in Equation (6). To evaluate and choose the best configuration, ShanghaiTech Part A is utilized because of the extremely congested scenes, the varied perspective, and unfixed resolution. To show the advantage of our proposed networks, they are evaluated the counting performance for testing estimation networks with pooling layers (Figure 3a), dilated convolutions (Figure 3b), and forward connections. For network with forward connections, the master network from Figure 4b was only utilized and modified by reserving the direction of a skip connection as calculated by Equation (13),

(13) $$Y_{L}=F_{L}\left(X_{L}\right)⊕F_{L-K}\left(X_{L-K}\right)$$

where $F_{L}$ is the *L*th of Conv blocks in estimation network with *K* forward connections. $X_{L}$ is assigned as their input feature maps and $Y^{{}_{L}}$ is the output feature map from $F_{L}$. Table 1 shows the performance of our estimation networks with various configuration, where backward consisting of K=1, 2, and 3. The empirical result shows that dilated convolution overcomes estimation networks with pooling layers. In overall case, the estimation network with a K=1 backward connection overcomes other configurations. Therefore, we decided to select this configuration for comparison with related work in the next subsection.

**Figure 6:**
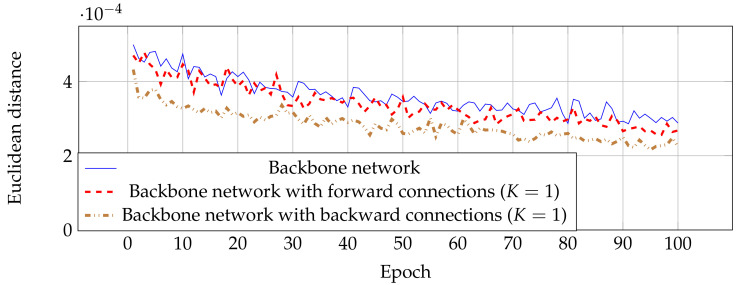
Learning curve between Euclidean distance and epoch while training the network.

Even though estimation networks with forward and backward connections have similar performance as shown in Table 1, the backward was designed for improving the estimation performance in an earlier stage. This means that this technique reaches the most optimal parameters faster than estimation networks without skip connections. Figure 6 shows the MSE from validated images in each epoch, where networks with backward connections have a low error at the beginning of training compared with the other two networks. This result indicates that estimation networks with backward connections can be trained faster by optimizing the feature map in shallow layers.

### 6.4. Counting Evaluation and Comparison

In this subsection, the proposed method was evaluated and compared with other related works using the evaluation metric described in Section 6.1. The experimental results were obtained from their configurations. Table 2 illustrates the counting performance of the proposed method and other estimation networks. The counting performance in each dataset are described as follows:

#### 6.4.1. Shanghaitech Dataset

The experimental process has analyzed this dataset into two groups (Part A and Part B). As shown in Table 2, the performance from estimation networks in Part A is worse than that in Part B because the crowd images obtained from part A have a wide range of a crowd density (from 0 to 1500 people) and image resolution. Therefore, the estimation network trained by a crowd image in part B can achieve more accurate results.

**Figure 7:**
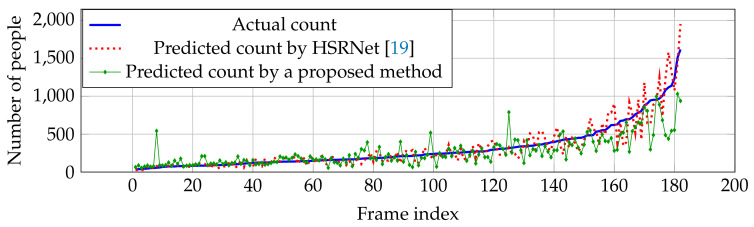
Actual and predicted counts with various crowd densities in ShanghaiTech dataset Part A.

**Figure 8:**
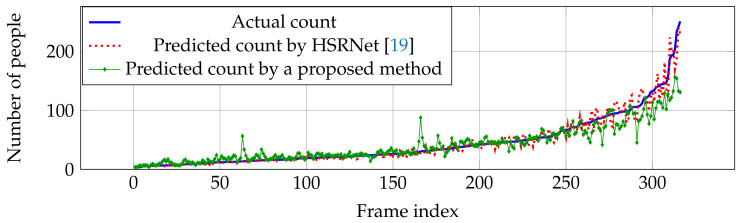
Actual and predicted counts with various crowd densities in ShanghaiTech dataset Part B.

Compared with other estimation networks, our performance cannot overcome other estimation networks, especially CP-CNN [18] and HSRNet [19]. Even though our result is not the best in the overall performance, our estimation network is more effective in high crowd density (>500 people), where HSRNet trends to have over-counting error shown in Figure 7. Similar with Part A, counting performance from Part B is effective in high crowd density, but obtain over-counting error from frame index 50 to 200 in Figure 8.

#### 6.4.2. UCF_CC_50

**Figure 9:**
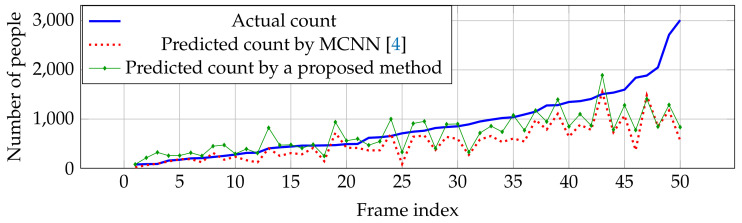
Actual and predicted counts with various crowd densities in UCF_CC_50 dataset.

As mentioned in Section 6.2.2, UCF_CC_50 contains a small number of crowd images. In the experiment, we utilized pre-trained weights from ShanghaiTech Part A for evaluating the counting performance in UCF_CC_50 because both crowd images in UCF_CC_50 and part A are obtained from the different websites, where they have various resolutions and lighting conditions to handle the testing data (UCF_CC_50). Table 2 shows that our performance outperforms other estimation networks in MAE, but obtain lower robustness in RMSE. In addition, they cannot handle a large number of people (>1500 people), as shown in Figure 9, which is out of range in crowd images obtained from the ShanghaiTech dataset in Part A.

#### 6.4.3. TRANCOS Dataset

**Figure 10:**
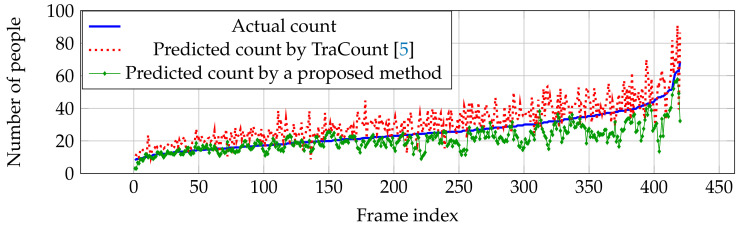
Actual and predicted counts with various crowd densities in TRANCOS dataset.

For vehicle counting, it clearly shows that our network outperforms other estimation networks in both RMSE and MAE as shown in Table 2. Since vehicle pattern might be similar to other objects (e.g., traffic light, building, and so on) in crowd images, most of the errors occur as over-counting from TraCount [5] in Figure 10. By knowing the characteristics of the target using the backward connection, false positives were reduced and an over-counting error can be solved by the proposed method as shown in Figure 10.

### 6.5. Density Map Assessment

The actual and predicted density maps were utilized for optimizing an estimation network. Predicting the density map and the number of objects in a crowd images. Due to this, the quality of a density map is the most important factor for counting performance. As far as we are aware, there are no standard rules to measure density map quality. In this paper, the predicted density map was evaluated by comparing with an actual density map and classifying the counting error which is either over-counting or under-counting error. The region of a predicted density map which has a higher or lower intensity than the actual density map is recognized as over-counting or under-counting error, respectively. Our predicted density was observed in different aspects described as follows:

#### 6.5.1. Object Scale and Crowd Density

**Figure 11:**
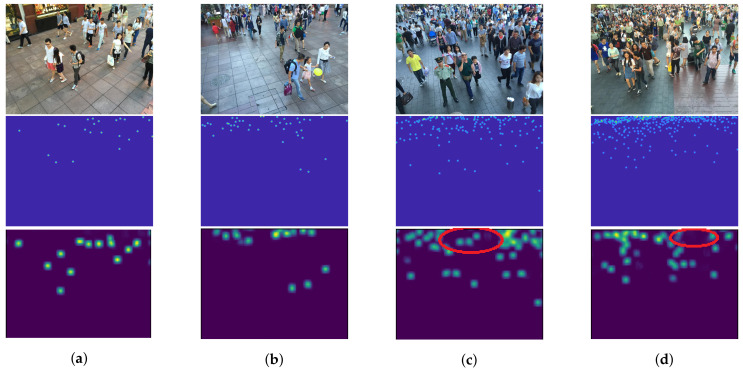
The examples of crowd images with different crowd density consisting of (**a**) 23, (**b**) 45, (**c**) 131, and (**d**) 297 people, where their input images, actual density maps, and predicted density maps by a proposed method are located in the 1st, 2nd, and 3rd rows, respectively.

As described in Section 1, we expected that the object size is proportional to the level of feature. Low and high-level features are considered as features of objects with small and large scales that are located in high and low crowd density, respectively. By improving low-level features with backward connections, a high-level feature can also be enriched because of a sequential structure of CNNs. Figure 11 shows examples of crowd images with their actual and predicted density map. The result shows that predicted density maps have a similar pattern with an actual density map in various crowd density, especially in low density (Figure 11a,b). However, our network is ineffective to objects with small scales causing the under-counting error as shown in Figure 11c,d within the red circles, because their resolutions might be too small for extracting their features or the number of their images are insufficient.

#### 6.5.2. Crowd Image Quality

**Figure 12:**
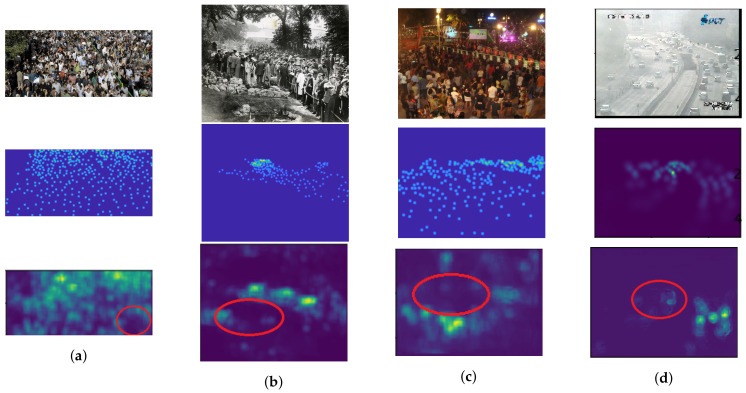
The examples of crowd images with counting errors caused by (**a**) small-sized crowd image, (**b**) object similarity, (**c**) dark illumination, and (**d**) occlusion of a mist, where their input images, actual density maps, and predicted density maps by a proposed method are located in the 1st, 2nd, and 3rd rows, respectively.

It is obvious that the quality of predicted density map depends on the quality of the input image. As described in Section 6.2, the crowd image might be obtained from several sources, similar with ShanghaiTech Part A and UCF_CC_50, which have various conditions (e.g., image resolution, camera viewpoints, and so on). In the previous section, it showed that our estimation network was not effective on their crowd images. The variation of image resolution creates an ambiguity between regions with high and low crowd density. Therefore, it causes under-counting and over-counting errors as shown in Figure 12a, in the red circle. In addition, over-counting error might be occurred when non-target objects are formulated in the predicted density map because of object similarity in different camera viewpoints as shown in Figure 12b. Since the crowd image is generally captured in the outdoor environment, the adverse condition are mainly concerned, where it causes an crowd image distortion in term of illumination and occlusion. As a result, objects located in this condition cannot be formulated in a predicted density map as shown in Figure 12c and Figure 12d by dark environment and occlusion from a mist, respectively, within red circles. The regions in these circles correspond as an under-counting error. In addition, the number of crowd images with adverse conditions in the experiment is insufficient. Then, their characteristics are hardly to predict the density map. It implied that our network is sensitive to a variation of image quality and its density distribution.

#### 6.5.3. Effect on Dilated Convolution

**Figure 13:**
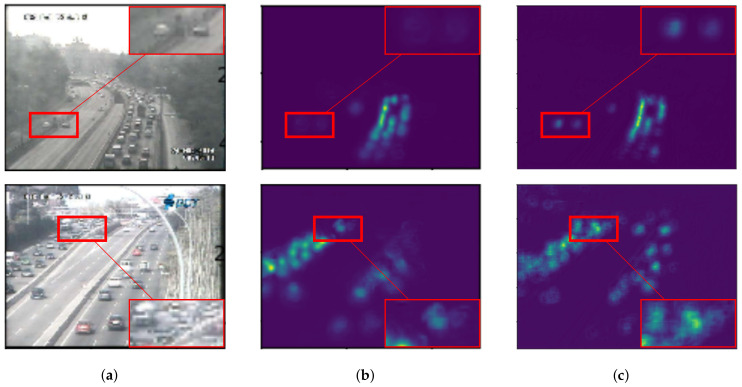
The example of effect on dilated convolution from vehicle counting where (**a**) input images and their prediceted density maps generated by the backbone network in (**b**) Figure 3a and (**c**) Figure 3b with backward connections.

**Figure 14:**
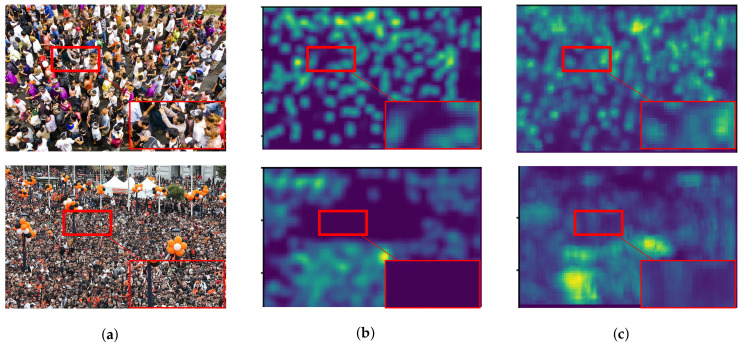
The example of effect on dilated convolution from people counting where (**a**) input images and their predicted density maps generated by the backbone network in (**b**) Figure 3a and (**c**) Figure 3b with backward connections.

With the advantage of a dilated convolution, it is expected to reduce information loss and increase image quality in a density map. To prove our hypothesis, we evaluated two backbone networks with and without dilated convolution as shown in Figure 3a,b, represented as network-*A* and network-*B*, respectively. Both of them were added to a backward connection with K=1 in Equation (6). In the experiment, network-*A* and network-*B* were tested for vehicle and people counting as shown in Figure 13 and Figure 14, respectively. These figures consist of input images (Figure 13a,b) and their predicted density maps produced by network-*A* (Figure 13b and Figure 14b) and network-*B* (Figure 13c and Figure 14c) with backward connections. For vehicle counting, empirical results show that dilated convolution generates more detail in predicted density, especially in high crowd density with small vehicles within red rectangular boxes. On the other hand, more people are formulated in predicted density by network-*B*. Therefore, it indicates that the error from under-counting error is reduced in the proposed method.

## 7. Conclusions and Future Work

In this paper, we have designed an estimation network to count objects in high crowd density. Our main contribution is the redesigned skip-network utilizing backward connections and dilated Conv layers. These connections help to improve the quality of feature maps in shallow layers by inserting the high-level features from deeper layers. In our expectation, non-target can be removed in the early stage by the enhancement in the shallow layer. Then an estimation network achieves better counting performance. On the other hand, a dilated Conv layer is introduced in skip-network to keep valuable information in high-level features, while the receptive field size can be exponentially increased as the function of pooling and up-sampling layers. The estimation network consists of slave and master networks for extracting high-level features and generating the final predicted density maps, respectively. In the experiment, the proposed method was evaluated in three datasets for counting people and vehicles. The estimation network with backward connection overcomes other configurations in counting performance. The empirical result shows that the proposed network is effective in high crowd density, where they provide more semantic detailed information by using dilated convolution. In addition, the proposed estimation network is able to remove false positive in density maps for reducing over-counting error, especially in TRANCOS dataset.

Even though our network obtained less counting error, it is ineffective on very high crowd density (>1500 people) increasing under-counting errors because of the low crowd image quality (e.g., resolution, distortion, and so on). As a result, the proposed method cannot overcome the performance of other estimation networks in the ShanghaiTech dataset and UCF_CC_50 containing the variation of image quality. On the other hand, our network still suffers from outdoor environments (e.g., lighting and weather conditions). According to these problems, it indicates that their number is insufficient for a deep learning technique. As future works, more datasets containing high crowd images (>1500 objects) and adverse conditions will be included to handle an object with small scale and image distortion. In addition, we plan to explore the routing algorithm for a skip connection for improving the quality of the predicted density map in various aspects.

## Figures and Tables

**Table 1:** The performance by the proposed estimation network with different configurations on ShanghaiTech Part A.

Estimation Network	RMSE	MAE
Estimation network with pooling layers (Figure 3a)	175.75	144.6
Backbone network (Figure 3b)	170.71	128.31
Backbone network with forward connections (K=1)	166.53	116.66
Backbone network with forward connections (K=2)	171.19	122.11
Backbone network with forward connections (K=3)	176.04	130.21
Backbone network with backward connections (K=1)	165.56	87.31
Backbone network with backward connections (K=2)	172.22	123.96
Backbone network with backward connections (K=3)	280.13	256.41

**Table 2:** Counting performance in ShanghaiTech, UCF_CC_50, and TRANCOS datasets.

Estimation Networks	ShanghaiTech A		ShanghaiTech B		UCF_CC_50		TRANCOS	
	RMSE	MAE	RMSE	MAE	RMSE	MAE	RMSE	MAE
MCNN [4]	173.2	110.2	41.1	26.2	509.1	377.6	18.28	11.05
CP-CNN [18]	106.2	73.6	30.1	20.1	-	-	-	-
Switching-CNN [2]	135	90.4	33.4	21.6	-	-	-	-
HSRNet [19]	100.3	62.3	11.8	7.2	-	-	-	-
Deep-stacked [20]	150.6	93.9	33.9	18.7	-	-	-	-
C-CNN [21]	141.7	88.7	22.1	14.9	-	-	-	-
Rodriguez et al. [22]	-	-	-	-	487.1	493.4	-	-
Lempitsky et al. [23]	-	-	-	-	541.6	419.5	-	-
Hydra net [1]	-	-	-	-	-	-	16.74	10.99
TraCount [5]	-	-	-	-	-	-	11.85	8.12
Proposed method	165.56	87.31	19.41	15.76	507.96	358.83	10.65	7.38
